# The relationship between empathic responses to a targeted anti-smoking campaign and smoke-free intention among adolescents: A cross-sectional analysis by smoking status

**DOI:** 10.18332/tid/240589

**Published:** 2026-10-21

**Authors:** A-Reum Jung, Guiohk Lee

**Affiliations:** 1 School of Media and Communication, Dankook UniversityYongin-siSouth Korea; 2 Department of Media and CommunicationSejong UniversitySeoulSouth Korea

**Keywords:** targeted anti-smoking message, empathy, adolescent, smoking status, smoke-free intention

## Abstract

**Introduction:**

Anti-smoking campaigns have been widely implemented to reduce adolescent smoking. However, these campaigns have heavily relied on fear appeals that emphasize the negative consequences of smoking. This study examined an alternative strategy based on empathy in a youth-targeted anti-smoking campaign. Specifically, it aimed to investigate the relationship between adolescents’ empathic responses to the campaign and their smoke-free intention, as well as the moderating role of smoking status in this relationship.

**Methods:**

This cross-sectional study examined the association between a multidimensional continuous independent variable (empathic responses, including empathic understanding, identification, and vicarious emotional response) and a continuous dependent variable (smoke-free intention), with smoking status (never smoked, susceptible to smoking, tried smoking, repeated smoking, and habitual smoking) as a categorical moderator. An online survey was conducted in South Korea from 26 January to 18 February 2021. A total of 2000 adolescents (1019 males and 981 females) completed a structured questionnaire assessing their empathic responses to the anti-smoking campaign, smoke-free intention, and smoking status.

**Results:**

The findings revealed that empathic responses to the anti-smoking campaign were positively associated with smoke-free intention, including empathic understanding (p<0.001), identification (p<0.001), and vicarious emotional response (p<0.001). These positive associations remained significant across most smoking status groups, including never smoked (p<0.001), susceptible to smoking (p<0.001), tried smoking (p<0.001), and repeated smoking (p<0.01). However, the strength of these associations decreased as adolescents progressed toward habitual smoking.

**Conclusions:**

The empathy-based anti-smoking campaign targeting adolescents was positively associated with adolescents’ smoke-free intention, although the strength of this relationship diminished as smoking became more habitual. These findings suggest that empathy-based anti-smoking campaigns could be an effective strategy for preventing smoking initiation among adolescents. However, additional strategies are required to promote smoking cessation among habitual adolescent smokers.

## Introduction

Both the mental and physical risks associated with smoking in adolescents are continuously reported^1^. Given adolescents’ heightened vulnerability to nicotine dependence and the likelihood of smoking persisting into adulthood^1^, their tobacco use warrants particular societal attention. However, strategies aimed at reducing smoking often fail to account for the unique characteristics of adolescents, focusing instead on the general population^2^. Moreover, anti-smoking campaigns targeting adolescents frequently continue to employ risk-focused messages that have been used repeatedly^3^.

South Korea has launched ‘NoDam Members’ (with ‘NoDam’ meaning ‘non-smoking’ in Korean) in 2020, the first nationwide anti-smoking campaign targeting adolescents^4^. The campaign promoted the message that choosing not to smoke is a positive and admirable behavior under peer pressure or temptation to smoke, reflecting personal strength and the ability to resist social pressures. Rather than relying primarily on fear-based appeals commonly used in anti-smoking campaigns^5^, the campaign sought to evoke empathy by portraying realistic and relatable scenarios^4,6^.

Empathy refers to the process through which individuals understand and relate to situations depicted in advertisements by projecting themselves into the narrative and sharing its emotional meaning^7^. Previous research indicates that empathy plays a critical role in generating positive advertising outcomes^8^. Advertisements that incorporate personally relevant elements – such as relatable experiences, imagery, or messaging – encourage deeper cognitive elaboration, which can lead to stronger persuasive effects^9^.

The primary objective of this study was to examine the relationship between empathy derived from everyday adolescent smoking-related experiences and smoke-free intention. This study is significant in that it evaluates targeted anti-smoking strategies for adolescents, distinguishing them from traditional adult-focused campaigns that predominantly rely on fear-based appeals.

## Methods

### Study design

This study employed a design shown in Figure 1, with a multidimensional continuous independent variable (empathic responses), a continuous dependent variable (smoke-free intention), and a multi-categorical moderator (smoking status).

**Figure 1:**
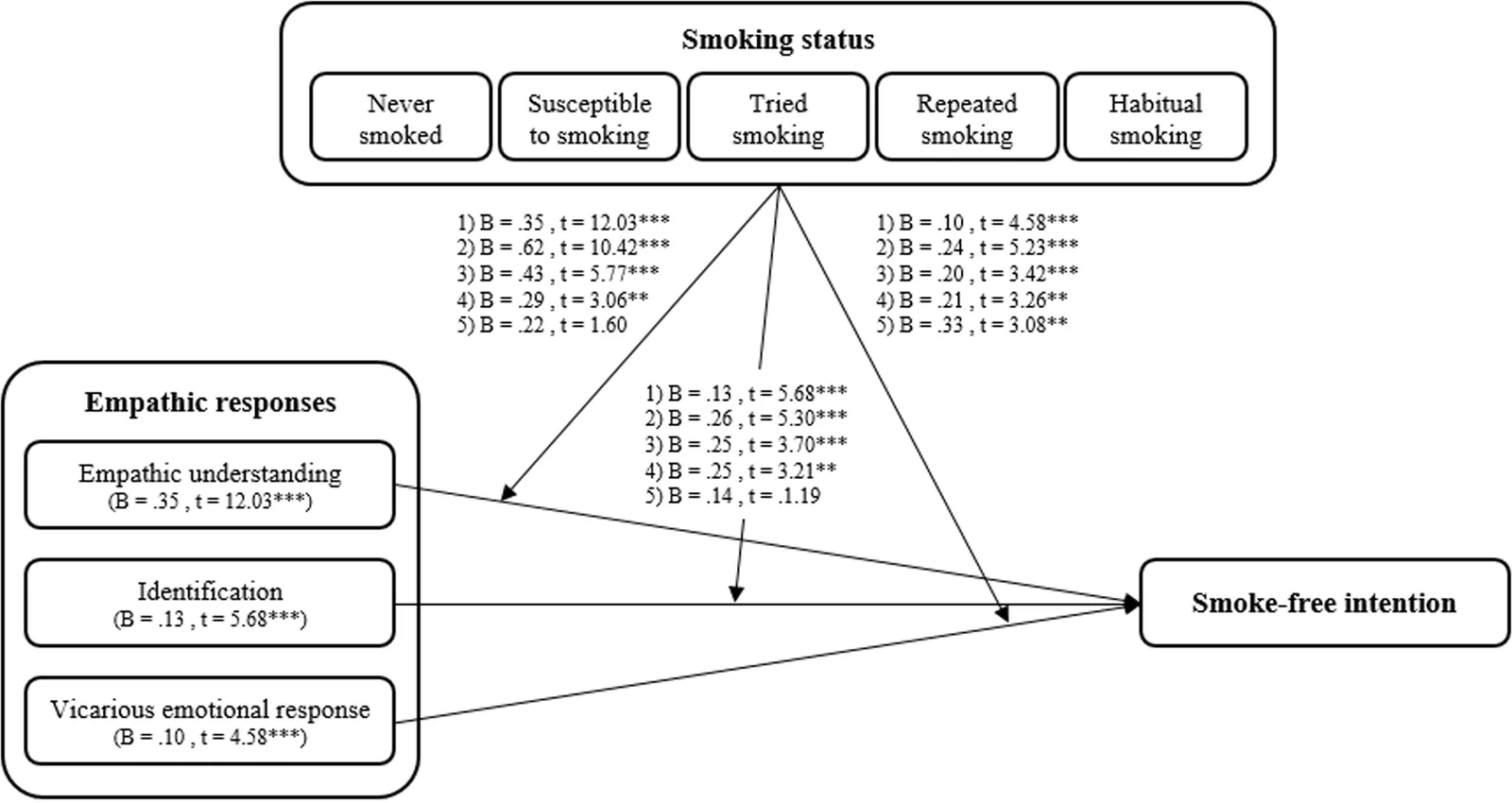
Study model

*p<0.05, **p<0.01, ***p<0.001.

#### Independent variable

First, the independent variable is empathic responses, which comprises three dimensions: empathic understanding, identification, and vicarious emotional response^7^.

- Empathic understanding: refers to the process of understanding the situation presented in the ad, along with the actions of the characters, and their underlying behavioral reasons, motivations, and emotions.
- Identification: refers to the engagement of comparing oneself to the characters in the ad and experiencing a sense of similarity between the character’s actions, reasons, motivations, and emotions and one’s own.
- Vicarious emotional response: refers to the experience of sharing the situation, challenges, desires, and emotions that the character encounters in the ad.

#### Moderation variable

Moderation variable is smoking status, which consists of five categories ranging from never smoked to habitual smoking. These categories were refined and operationalized based on the adolescent smoking stages developed by the Korea Ministry of Health and Welfare and the Korea Health Promotion Institute^10^.

- The never smoked group: individuals who have never smoked and have not experienced any desire or urge to smoke.
- The susceptible to smoking group: individuals who have never smoked, but experience the urge to smoke when exposed to smoking behavior by acquaintances or when smoking scenes are depicted in the media.
- The tried smoking group: individuals who have tried smoking, typically due to natural exposure in social contexts, such as when interacting with acquaintances.
- The repeated smoking group: individuals who smoke on a recurring basis under specific circumstances, such as in social settings with friends who smoke or in response to stress, but are able to function without smoking in their daily life.
- The habitual smoking group: individuals who smoke regularly, typically on a daily basis, and experience withdrawal symptoms when attempting to reduce the amount of smoking or when trying to quit altogether.

#### Dependent variable

The dependent variable is smoke-free intention, which refers to the intention to quit smoking for smokers and the intention to remain smoke-free for non-smokers.

#### Control variable

The level of ad exposure to the anti-smoking campaign across four media channels, including television, radio, outdoor, and online, was included as a control variable.

### Sample

An online survey was conducted in South Korea from 26 January to 18 February 2021. A total of 2198 adolescents participated in the survey, which was managed by the research firm EMBRAIN. Of these, 198 respondents who were not exposed to the anti-smoking campaign were excluded, resulting in a final sample of 2000 respondents. A stratified random sampling method was used to ensure representative distribution to the total adolescent population of South Korea.

### Procedure

Adolescents who consented to participate received instructions before completing a structured questionnaire which included items on the amount of ad exposure, empathic responses to the ad, smoke-free intention, smoking status, and demographic information. Respondents who reported no exposure to the campaign were directed to skip the questions related to the ad.

### Measurements

Empathic responses were measured across three dimensions: empathic understanding, identification, and vicarious emotional response^7^.

Empathic understanding (a=0.88) was assessed using four items including: ‘While watching the ad, I understand the events as they occurred’, ‘While watching the ad, I understood what the characters’ needs’, ‘While watching the ad, I understand the characters’ motivation for the behavior’, and ‘While watching the ad, I understood what the characters were feeling’.

Identification (a=0.89) was measured using four items including ‘While watching the advertisement, I felt that the events portrayed in the ad were similar to events I have experienced’, ‘Watching the advertisement, I felt that the feelings expressed by the characters in the ad were similar to feelings I have felt’, ‘While watching the advertisement, I felt that the needs of the characters in the ad were similar to my own needs’, and ‘While watching the advertisement, I felt that similar events could occur to me in the future’.

Vicarious emotional response (a=0.93) was measured using three items including ‘While watching the ad, I experienced feeling as if the events were really happening to me’, ‘While watching the ad, I felt as though I were one of the characters’, and ‘While watching the ad, I felt as though the events in the ad were happening to me’. All measures of empathic responses were assessed using a 5-point Likert scale (1=strongly disagree to 5=strongly agree).

Smoke-free intention (a=0.83)^11^ was measured using three items, including ‘After watching the ad, I want to encourage others to quit smoking’, ‘After watching the ad, I have no plans to smoke in the future’, and ‘After watching the ad, I lost interest in smoking’, with a 5-point Likert scale (1=strongly disagree to 5=strongly agree).

Exposure to the anti-smoking campaign was assessed using a self-report 5-point Likert scale (1=not at all to 5=a lot). Respondents indicated their exposure to the campaign across four types of media: television, radio, outdoor, and online.

### Statistical analysis

The data were analyzed using the SPSS PROCESS macro model number 1. The three dimensions of empathic responses served as independent variables, while smoke-free intention was the dependent variable. Smoking status was included as a categorical moderator. The mean value of ad exposure across the four media types was controlled, based on previous research indicating a significant relationship between exposure to anti-smoking campaigns and smoking behavior^12^.

The SPSS PROCESS macro provides regression-based estimates^13^. In this analysis, empathic responses were regressed on smoke-free intention. Additional *post hoc* analyses were conducted to examine the relationship between empathic responses and smoke-free intention within each smoking status. Statistical significance was determined as p<0.05.

## Results

Of the 2000 respondents, 50.9% (n=1019) were male, and 49.4% (n=981) were female. The mean age of the participants was 16.26 years (SD=1.25; range: 14–18) Smoking status was categorized into five groups: never smoked (n=1298; 64.9%), susceptible to smoking (n=287; 14.4%), tried smoking (n=202; 10.1%), repeated smoking (n=148; 7.4%), and habitual smoking (n=65; 3.3%).

The results indicated that all three types of empathic responses – empathic understanding (B=0.35, SE=0.03, t=12.03, p<0.001; 95% CI: 0.30–0.41), identification (B=0.13, SE=0.03, t=5.68, p<0.001; 95% CI: 0.08–0.17), and vicarious emotional response (B=0.10, SE=0.02, t=4.58, p<0.001; 95% CI: 0.05–0.14) – were positively associated with smoke-free intention.

The relationship between empathic responses and smoke-free intention varied according to smoking status. Specifically, the relationship between empathic understanding and smoke-free intention differed significantly between the susceptible to smoking group and the never smoked group (B=0.26, SE=0.07, t=4.01, p<0.001; 95% CI: 0.13–0.39). *Post hoc* analysis indicated that empathic understanding was positively associated with smoke-free intention among the never smoked group (B=0.35, SE=0.03, t=12.03, p<0.001; 95% CI: 0.30–0.41), the susceptible to smoking group (B=0.62, SE=0.06, t=10.42, p<0.001; 95% CI: 0.50–0.73), the tried smoking group (B=0.43, SE=0.07, t=5.77, p<0.001; 95% CI: 0.28–0.58), and the repeated smoking group (B=0.29, SE=0.10, t=3.06, p<0.01; 95% CI: 0.11–0.48). However, this association was not significant in the habitual smoking group (B=0.22, SE=0.14, t=1.61, p>0.05; 95% CI: -0.05–0.50).

Similarly, the relationship between identification and smoke-free intention differed significantly between the susceptible to smoking group and the never smoked group (B=0.13, SE=0.05, t=2.47, p<0.05; 95% CI: 0.03–0.24). *Post hoc* analysis further revealed that identification was positively associated with smoke-free intention among the never smoked group (B=0.13, SE=0.02, t=5.68, p<0.001; 95% CI: 0.08–0.17), the susceptible to smoking group (B=0.26, SE=0.06, t=5.30, p<0.001; 95% CI: 0.17–0.36), the tried smoking group (B=0.25, SE=0.07, t=3.70, p<0.001; 95% CI: 0.12–0.38), and the repeated smoking group (B=0.25, SE=0.08, t=3.21, p<0.001; 95% CI: 0.10–0.39). In contrast, the association was not significant in the habitual smoking group (B=0.14, SE=0.12, t=1.19, p>0.05; 95% CI: -0.09–0.38).

Additionally, the relationship between vicarious emotional response and smoke-free intention differed significantly between the susceptible to smoking group and the never smoked group (B=0.14, SE=0.05, t=2.86, p<0.01; 95% CI: 0.04–0.24), as well as between the habitual smoking group and the never smoked group (B=0.23, SE=0.10, t=2.15, p<0.05; 95% CI: 0.02–0.45). *Post hoc* analysis indicated that vicarious emotional response was positively associated with smoke-free intention across all smoking-status groups, including the never smoked group (B=0.10, SE=.02, t=4.58, p<0.001; 95% CI: 0.05–0.14), the susceptible to smoking group (B=0.24, SE=0.05, t=5.23, p<0.001; 95% CI: 0.15–0.33), the tried smoking group (B=0.20, SE=0.06, t=3.42, p<0.001; 95% CI: 0.08–0.31), the repeated smoking group (B=0.21, SE=0.07, t=3.26, p<0.01; 95% CI: 0.08–0.34), and the habitual smoking group (B=0.33, SE=0.11, t=3.08, p<0.01; 95% CI: 0.12–0.54). Detailed statistical results are presented in Table 1.

**Table 1:** The relationship between empathic responses to the targeted anti-smoking campaign and smoke-free intention across smoking status

	B	SE	t	LLCI	ULCI
***Empathic understanding***	0.35	0.03	12.03***	0.30	0.41
**Smoking status** (vs never smoked: 0)					
Susceptible	-1.36	0.26	-5.26***	-1.87	-0.85
Tried	-0.96	0.32	-3.02**	-1.58	-0.33
Repeated	-1.09	0.40	-2.77**	-1.87	-0.32
Habitual	-1.10	0.55	-2.01*	-2.18	-0.02
**Empathic × smoking status** (vs never smoked: 0)					
Empathic × susceptible	0.26	0.07	4.01***	0.13	0.39
Empathic × tried	0.08	0.08	0.97	-0.08	0.23
Empathic × repeated	-0.06	0.10	-0.59	-0.26	0.14
Empathic × habitual	-0.13	0.14	-0.91	-0.41	0.15
Exposure	0.12	0.03	4.84***	0.07	0.17
**Conditional effect**					
Never smoked	0.35	0.03	12.03***	0.30	0.41
Susceptible	0.62	0.06	10.42***	0.50	0.73
Tried	0.43	0.07	5.77***	0.28	0.58
Repeated	0.29	0.10	3.06**	0.11	0.48
Habitual	0.22	0.14	1.61	-0.05	0.50
***Identification***	0.13	0.03	5.68***	0.08	0.17
**Smoking status** (vs never smoked: 0)					
Susceptible	-0.82	0.18	-4.53***	-1.17	-0.46
Tried	-1.13	0.25	-4.51***	-1.62	-0.64
Repeated	-1.85	0.30	-6.14***	-2.45	-1.26
Habitual	-1.74	0.45	-3.91***	-2.61	-0.87
**Identification × smoking status** (vs never smoked: 0)					
Identification × susceptible	0.13	0.05	2.47*	0.03	0.24
Identification × tried	0.12	0.07	1.68	-0.02	0.25
Identification × repeated	0.12	0.08	1.47	-0.04	0.27
Identification × habitual	0.02	0.12	0.13	-0.23	0.26
Exposure	0.18	0.03	6.78***	0.13	0.23
**Conditional effect**					
Never smoked	0.13	0.02	5.68***	0.08	0.17
Susceptible	0.26	0.05	5.30***	0.17	0.36
Tried	0.25	0.07	3.70***	0.12	0.38
Repeated	0.25	0.08	3.21**	0.10	0.39
Habitual	0.14	0.12	1.19	-0.09	0.38
***Vicarious emotional response***	0.10	0.02	4.58***	0.05	0.14
**Smoking status** (vs never smoked: 0)					
Susceptible	-0.75	0.14	-5.27***	-1.03	-0.47
Tried	-1.02	.20	-5.19***	-1.40	-0.63
Repeated	-1.78	0.23	-7.64***	-2.24	-1.32
Habitual	-2.40	0.36	-6.75***	-3.10	-1.70
**Vicarious × smoking status** (vs never smoked: 0)					
Vicarious × susceptible	0.14	0.05	2.86**	0.04	0.24
Vicarious × tried	0.10	0.06	1.66	-0.02	0.22
Vicarious × repeated	0.12	0.07	1.72	-0.02	0.25
Vicarious × habitual	0.23	0.11	2.15*	0.02	0.45
Exposure	0.18	0.03	6.94***	0.13	0.23
**Conditional effect**					
Never smoked	0.10	0.02	4.58***	0.05	0.14
Susceptible	0.24	0.05	5.23***	0.15	0.33
Tried	0.20	0.06	3.42***	0.08	0.31
Repeated	0.21	0.07	3.26**	0.08	0.34
Habitual	0.33	0.11	3.08**	0.12	0.54

SE: standard error. LLCI: lower limit of 95% confidence interval. ULCI: upper limit of 95% confidence interval. A total of 2000 adolescents responded to an online survey conducted in South Korea from 26 January to 18 February 2021.

*p<0.05, **p<0.01, ***p<0.001**.**

## Discussion

This study aimed to investigate the association between adolescents’ empathic responses to an anti-smoking campaign targeting adolescents and their smoke-free intention, as well as to examine whether this relationship was moderated by smoking status. The findings indicate that empathic responses were positively associated with adolescents’ smoke-free intention, indicating that empathy-based messages targeting adolescents can effectively prevent smoking initiation among non-smoking adolescents and encourage smoking cessation in adolescents who smoke. However, the strength of the positive relationship between empathic responses and smoke-free intention varied depending on smoking status. The strength of this association decreased as smoking became more habitual.

The favorable effectiveness of anti-smoking campaigns has been widely documented^12,14,15^. Importantly, this study extends previous findings by highlighting the value of targeted strategies in an anti-smoking campaign. Ads that include self-relevant content encourage individuals to engage with and process the messages, resulting in more persuasive outcomes^9^. A deeper understanding of the adolescent context is critical for developing effective strategies to reduce smoking behaviors. Further refinement of ad messages tailored to smoking status separately is essential, given the important role that smoking status plays in shaping response to anti-smoking messages.

In this study, empathic responses were particularly effective among adolescents who had not yet smoked, including those in the susceptible to smoking group. Because smokers, particularly those who began smoking at an early age, often face significant challenges in quitting due to various reasons such as nicotine dependence^1,16-18^, it is critical to prevent adolescents from initiating smoking so that the campaign can effectively act. Additionally, as studies have shown that adolescents progress to regular smoking, it becomes more difficult for anti-smoking campaigns to produce significant smoke-free intention. To encourage cessation among habitual smokers, a deeper level of empathic response, such as vicarious emotional responses, may be required. While preventing adolescent smoking initiation remains a crucial strategy, more targeted interventions are required for people who smoke, particularly habitual smokers showing greater resistance to quitting.

### Limitations

Although this study provides valuable insights into adolescent-targeted anti-smoking strategies, future research should explore additional factors that may influence smoking behaviors. First, further research on target-specific elements of anti-smoking campaigns, including the role of media characteristics and usage patterns, contributes to the development of more effective campaigns. Second, methodological improvement should be considered. Because cross-sectional studies cannot establish causal relationships, future research needs to employ study designs that allow stronger cause-and-effect inferences. Additionally, data collection methods that reduce information biases and misclassification related to self-reported measures should be considered. Finally, this study was conducted in South Korea. Although this study included a sufficiently large sample size drawn from a nationally representative population, specific social and cultural contexts of South Korea may limit the generalizability of the findings. Future studies conducted across different countries and cultural contexts are needed to validate and extend the findings of this study.

## Conclusions

This study demonstrates the significance of empathy-based targeted messages in anti-smoking campaigns. In particular, the findings indicate that communicating the negative consequences of smoking, such as risk-based fear appeal strategies, is not the only effective approach for decreasing smoking-related behaviors. Rather, emphasizing positive aspects – such as framing the decision not to smoke as an admirable behavior that resists peer and social pressure – may serve as an effective strategy for promoting smoking prevention and cessation by eliciting empathic responses to the situations depicted in the campaign. However, the results also suggest that the effectiveness of anti-smoking campaigns varies depending on smoking status, even when target-specific empathic strategies are employed. As adolescents become habitual smokers, they become less responsive to the messages and maintain the intention to continue smoking. Additional strategies that consider not only age but also individuals’ smoking status are required to effectively promote smoking cessation among habitual adolescent smokers.
